# Correlation of ABO blood groups with treatment response and efficacy in infants with persistent pulmonary hypertension of the newborn treated with inhaled nitric oxide

**DOI:** 10.1186/s12884-023-05558-w

**Published:** 2023-04-22

**Authors:** Yi Guan, Ya Jin, Yongxue Lu, Dang Ao, Pingjiao Gu, Jiyan Yang, Guosheng Liu, Shasha Han

**Affiliations:** 1 Department of Pediatrics and Neonatology, Institute of Fetal-Preterm Labor Medicine, The First Affiliated Hospital of Jinan University, No.601 Huangpu Road West, Guangzhou, 510630 China; 2 The First People’s Hospital of Foshan, Foshan, 528010 China; 3 Department of Neonatology, the Affiliated Hospital of Guangdong Medical University, Zhanjiang, 524001 China; 4 Neonatology Department of Foshan Women and Children Hospital, Foshan, 528099 China; 5 Neonatology Department, Guangdong Women and Children Hospital, Guangdong Neonatal ICU Medical Quality Control Center, Guangzhou, 511442 China

**Keywords:** ABO blood groups, Efficacy, Inhaled nitric oxide, Persistent pulmonary hypertension of the newborn, Response

## Abstract

**Objective:**

Not all infants with persistent pulmonary hypertension of the newborn (PPHN) respond to inhaled nitric oxide (iNO) therapy, as it is known to improve oxygenation in only 50% to 60% of cases. In this study, we investigated whether ABO blood groups were a relevant factor affecting the improvement of oxygenation by nitric oxide (NO) therapy in infants with PPHN.

**Methods:**

This study was a retrospective, multicenter, and cohort-controlled trial that involved 37 medical units. Infants with PPHN who met the inclusion criteria and were treated with NO (a vasodilator) alone from July 1, 2015, to June 30, 2020, were selected and assigned into three groups: blood type A, blood type B, and blood type O (there were only 7 cases of blood type AB, with a small number of cases, and therefore, blood type AB was excluded for further analysis). The response to iNO therapy was defined as an increase in the ratio of the partial pressure of arterial oxygen (PaO2)/fraction of inspired oxygen (FiO2) > 20% from the basal value after treatment. Oxygenation was assessed mainly based on the two values, oxygenation index (OI) and PaO2/FiO2. The correlation of ABO blood groups with responses to iNO therapy and their influence on the efficacy of iNO therapy was analyzed based on the collected data.

**Results:**

The highest proportion of infants with PPHN who eventually responded to iNO therapy was infants with blood type O. Infants with blood type O more readily responded to iNO therapy than infants with blood type B. Oxygenation after iNO treatment group was optimal in the blood type O group and was the worst in the blood type A group among the three groups. Infants with blood type O showed better efficacy than those with blood types A and B.

**Conclusion:**

ABO blood groups are correlated with responses to iNO therapy in infants with PPHN, and different blood groups also affect the efficacy of NO therapy in infants with PPHN. Specifically, infants with blood type O have a better response and experience the best efficacy to iNO therapy.

## Introduction

Persistent pulmonary hypertension of the newborn (PPHN) is a condition wherein there is right-to-left shunting of blood at the atrial and/or arterial ductal level in newborns secondary to the failure of fetal-normal “adult” circulation transition, caused by a persistent increase in pulmonary vascular resistance after birth, which is clinically manifested as severe hypoxemia [1, 2]. The incidence of PPHN is approximately 0.2% and reaches 2% in very low-birth-weight infants [3], while going up to 10% in all newborns with respiratory failure suffering from pulmonary arterial hypertension [4]. Inhaled nitric oxide (iNO) improves oxygenation and reduces the need for extracorporeal membrane oxygenation (ECMO) in near full-term and full-term infants with PPHN. Nitric oxide (NO) has been used for clinically treating pulmonary arterial hypertension since the 1980s. Furthermore, iNO was approved by the U.S. Food and Drug Administration (FDA) in 1999 and by the European Medicines Agency (EMA) in 2001 as a routine treatment for infants with PPHN at 34 weeks of gestational age or older [5]. Nevertheless, not all infants treated with iNO present with improved oxygenation. Relevant randomized controlled trials showed that only 50%–60% of infants responded to iNO therapy [6–9]. It remains unclear which factors influence the response and efficacy of iNO in infants with PPHN. In a randomized trial by Kinsella et al. [10], the efficacy in infants treated with and without iNO was compared, which revealed that only 123 infants with PPHN (60%) responded to single or combined treatment. In contrast, 40% of infants did not respond to high-frequency oscillatory ventilation and/or iNO. Weimann et al. observed that responses to iNO therapy were weaker in adults of blood type B/AB with acute respiratory distress syndrome (ARDS) than in adults of blood type O/A [11]. Many factors have been reported to affect the efficacy of iNO therapy, one of which may be different ABO blood groups. According to related literature [12], the response to iNO therapy is defined as a > 20% increase in the ratio of the partial pressure of arterial oxygen (PaO2)/fraction of inspired oxygen (FiO2) from the basal value after treatment. This criterion was used in this study to analyze the correlation of blood groups with responses to iNO therapy and their influence on the efficacy of iNO therapy in infants with PPHN. We hypothesized that different blood types might affect the efficacy of iNO in children with PPHN, with children with O blood type possibly having a more pronounced effect on the treatment outcome.

## Material and methods

### Study population

The inclusion criteria of patients were as follows: infants who were diagnosed with PPHN based on the clinical and ultrasound diagnostic criteria for PPHN from July 1, 2015, to June 30, 2020, and hospitalized for NO therapy for at least 72 h.

In this multicenter retrospective study, we included 90 infants with PPHN who met the inclusion criteria without congenital heart disease, congenital diaphragmatic hernia, or missing medical records and were treated with NO (treatment with NO alone without other vasodilators), of whom 24 (26.7%) were of blood type A, 20 (22.2%) of blood type B, 39 (43.3%) of blood type O, and 7 (7.8%) of blood type AB. As there were very few cases of blood type AB—only 7, blood type AB was excluded from further analysis. The 83 children with PPHN who were included in the further analysis were allocated into three groups: blood type A, blood type B, and blood type O for the related study.

### Diagnosis of PPHN

The clinical diagnostic criteria of PPHN [13, 14] were as follows: ① 5% or more difference in percutaneous oxygen saturation (SaO2) (SaO2 values in the lower extremity were lower than those in the right upper extremity) or differences of 10–20 mmHg between PaO2 values before (right upper extremity) and after (lower extremity) opening of ductus arteriosus; ② significant hypoxemia, with no improvement in oxygen saturation after oxygen inhalation with a hood or hyperoxic ventilation; ③ disproportion between the manifestation of hypoxia and the degree of lung disease shown on X-ray; ④ PPHN with an onset of less than 1 week or PPHN where ECMO or 2 weeks of conventional treatment was ineffective. The ultrasound diagnostic criteria of PPHN [15, 16] were as follows: ① systolic pulmonary arterial pressure > 35 mmHg or > 2/3 systolic blood pressure of systemic circulation; ② existence of right-to-left shunting of blood at the atrial and/or arterial ductal level.

### Definition

The criterion to establish an eventual iNO response was over 20% increase in PaO2/FiO2 after treatment compared to the baseline value [12, 17]. It was the same for all medical units involved in this study.

Oxygenation index (OI) is a target in the treatment of acute lung injury, acute respiratory distress syndrome (ARDS), and PPHN [18], which is an essential index of the availability of sufficient oxygen to organs and tissues for oxygenation to obtain energy, as well as an index to assess the severity of the disease and to judge the disease outcome. In the neonatal intensive care unit, OI is commonly used to evaluate the severity of hypoxic respiratory failure (HRF) and PPHN in newborns [19]. OI is calculated using the following formula: OI = FiO2 * mean airway pressure * 100/PaO2. PaO2/FiO2, a more clinically accessible index, has been proposed in the literature as a criterion for determining efficacy in patients undergoing PPHN treatment [20].

Therefore, these two indexes were utilized in this study to analyze the response and efficacy of NO in PPHN infants with different blood types.

### Data collection

**Fig. 1:**
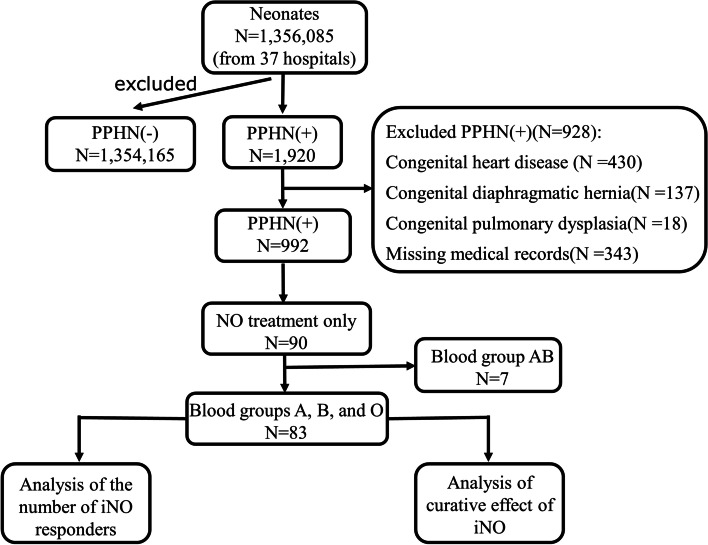
Flow chart of the study

Thirty-seven medical units in Guangdong Province formed the Multicenter Research Collaborative Group for PPHN. A total of 1,356,085 newborns were born in these 37 hospitals from July 1, 2015, to June 30, 2020, among which 1,920 were diagnosed with PPHN and 992 met the inclusion criteria for this study. Furthermore, 90 infants with PPHN were treated with only one vasodilator, NO, and there were 83 infants with blood types A, B, and O. Each member of the collaborative group arranged personnel to collect the cases of infants with blood types A, B, and O who responded to iNO therapy, as well as the basic information of PPHN infants treated with iNO, the results of blood gas analysis (PH, PaO2, partial pressure of carbon dioxide [PaCO2], and SaO2) and mechanical ventilation parameters (FiO2 and mean arterial pressure [MAP]) before treatment and after 24–48 h of treatment, 72 h of treatment, and termination of treatment (first detection after the termination of treatment). OI and PaO2/FiO2 values were calculated based on the results of blood gas analysis and mechanical ventilation parameters to analyze the correlation of ABO blood groups with the response and efficacy of iNO therapy (Fig. 1).

### Statistical analysis

The data were tested for normality. Normally distributed measurement data are expressed as mean ± standard deviation ($\overline{\mathrm x}$ ± s), and the *t*-test and analysis of variance were used for comparisons between groups. Non-normally distributed measurement data were summarized as M (Q1-Q3), and the rank sum test was used to compare groups. Count data are presented as cases (%), and the χ^2^ test was used to compare groups. *P* < 0.05 was considered a statistically significant difference. All data were processed and analyzed with SPSS 23.0 statistical software.

### Ethics

This study was approved by the Institutional Review Committee. As this retrospective study did not fall under the category of *Medical Research Involving Human Subjects*, informed consent was not required as we used anonymous clinically obtained data.

## Results

### Comparison of basic information among the three groups

The basic information after birth, including sex, gestational age, birth weight, 5-min Apgar scores, and PaO2/FiO2, were analyzed in infants with PPHN of blood type A, blood type B, and blood type O groups. The results revealed no statistically significant differences in sex (χ^2^ = 0.658, *P* = 0.720), gestational age (*F* = 0.811, *P* = 0.448), 5-min Apgar scores (*F* = 0.405, *P* = 0.669), birth weight (H = 1.221, *P* = 0.543), and PaO2/FiO2 (H = 2.749, *P* = 0.253) among the three groups (*P* > 0.05; Table 1). Also, there was no significant difference in the mean age at admission among the three groups (Blood type A: 20.375 h, Blood type B 50.975 h, and Blood type O: 34.829 h; *P* > 0.05).

**Table 1:** Basic information of PPHN infants with ABO blood groups

Items	Blood type A (*n* = 24)	Blood type B (*n* = 20)	Blood type O (*n* = 39)	F/χ^2^	*P*
Sex: male/female	15/9	13/7	28/11	0.658	0.720
Gestational age (weeks)	36.4 ± 3.9	34.8 ± 4.9	35.9 ± 4.1	0.811	0.448
Birth weight (g)	2925.0(1852.5–3300.0)	2610.0(1612.5–3065.0)	2800.0(2270.0–3200.0)	1.221^a^	0.543
5-min Apgar scores	8.8 ± 2.1	8.6 ± 2.4	9.1 ± 1.6	0.405	0.669
PaO2 /FiO2	120.0(86.1–160.0)	168.5(108.2–279.7)	127.5(86.3–250.7)	2.749^a^	0.253

^a^Is the statistic of the Kruskal Wallis test

### Analysis of the number of infants responding to iNO therapy in the three groups

A total of 83 PPHN infants with blood types A, B, and O were treated with NO, including 24 in the blood type A group, 20 in the blood type B group, and 39 in the blood type O group. The number of infants with PPHN responding to iNO therapy after 24 to 48 h of treatment was statistically analyzed in the three groups. The results demonstrated that the difference was not statistically significant (χ^2^ = 4.267, *P* = 0.118). The number of infants with PPHN responding to iNO therapy after 72 h of treatment was statistically analyzed in the three groups. It was statistically significantly different among the three groups (χ^2^ = 10.477, *P* = 0.005). No significant difference was observed in the number of infants with PPHN responding to iNO therapy between the blood type A and blood type O groups.

In contrast, a significant difference was found between these two groups and the blood type B group, with the fewest infants with PPHN responding to iNO therapy in the blood type B group (7 cases, 35%). After the cessation of treatment, the number of infants with PPHN responding to iNO therapy exhibited a statistical difference among the three groups (χ^2^ = 10.188, *P* = 0.006). The number of infants with PPHN responding to iNO therapy was statistically significantly higher in the blood type O group (34 cases, 87.2%) than in the blood type B group but was not significantly different between these two groups and the blood type A group (Table 2).

**Table 2:** Analysis of the number of infants with responses in the three groups

Blood groups	Total number of infants	24–48 h of treatment Number of infants responding to iNO therapy (%)	72 h of treatment Number of infants responding to iNO therapy (%)	After the termination of treatment Number of infants responding to iNO therapy (%)
Blood type A	24	13 (54.2)	18_a_ (75.0)	19 _a, b_ (79.2)
Blood type B	20	5 (25.0)	7_b_ (35.0)	10 _b_ (50.0)
Blood type O	39	19(48.7)	29_a_ (74.4)	34_a_ (87.2)
χ^2^	-	4.267	10.477	10.188
P	-	0.118	0.005	0.006

The same subscript letter in the table indicates no difference between groups, while different subscript letters indicate significant differences between groups

Among infants with the three different blood types, A, B, and O, the highest proportion of PPHN infants who eventually responded to iNO therapy was infants with blood type O; infants with blood type O more readily responded to respond to iNO therapy than infants with blood type B.

### Analysis of the efficacy in the three groups

Before treatment, OI (H = 2.333, *P* = 0.311) and PaO2/FiO2 (H = 2.749, *P* = 0.253) were not statistically significantly different among the three groups (*P* > 0.05). From 24 to 48 h of treatment, differences in OI (H = 3.175, *P* = 0.204) and PaO2/FiO2 (H = 3.700, *P* = 0.157) were not statistically significant among the three groups (*P* > 0.05). At 72 h of treatment, no statistically significant difference was found in OI (H = 3.362, *P* = 0.186) and PaO2/FiO2 (H = 2.799, *P* = 0.247) among the three groups (*P* > 0.05). After the treatment was stopped, OI (H = 8.851, *P* = 0.012) values were statistically significantly lower, but PaO2/FiO2 values (H = 9.395, *P* = 0.009) were statistically significantly higher in the blood type O group than in the blood type A and blood type B groups (*P* < 0.05).

Pairwise comparisons were conducted among the three groups after the treatment was stopped. The results demonstrated a difference in OI between the blood type B and blood type O groups (adjusted *P* = 0.030) and between the blood type A and blood type O groups (adjusted *P* = 0.043) but no difference in OI between the blood type A and blood type B groups (adjusted *P* = 1.000). For PaO2/FiO2, a difference was found between the blood type B and blood type O groups (adjusted *P* = 0.022) and blood type A and blood type O groups (adjusted *P* = 0.038). In contrast, there was no significant difference in PaO2/FiO2 between the blood type A and blood type B groups (adjusted *P* = 1.000).

After iNO treatment, oxygenation was the best in the blood type O group, but the poorest in the blood type A group among the three groups. In addition, the efficacy in infants with blood type O was more potent than in infants with blood types A and B (Table 3).

**Table 3:** Comparison of OI and PaO2/FiO2 before and after treatment

Items	Blood type A	Blood type B	Blood type O	H	*P*
Before treatment					
OI	12.3(8.7–19.8)	7.8(4.5–14.0)	10.9(4.9–18.9)	2.333	0.311
PaO2/FiO2	120.0(86.1–160.0)	168.5(108.2–279.7)	127.5(86.3–250.7)	2.749	0.253
24–48 h of treatment					
OI	11.6(6.0–18.2)	14.9(12.3–21.3)	8.1(4.2–19.0)	3.175	0.204
PaO2/FiO2	138.2(86.9–225.9)	95.8(75.0–132.0)	180.3(89.8–270.6)	3.700	0.157
72 h of treatment					
OI	6.3(5.5–13.7)	13.2(6.5–15.8)	6.5(3.8–8.3)	3.362	0.186
PaO2/FiO2	221.4(102.5–250.0)	113.5(82.2–201.7)	195.8(148.2–303.4)	2.799	0.247
After the termination of treatment					
OI	7.8(3.9–8.6)	6.1(5.3–11.2)	3.9(2.6–4.7) *	8.851	0.012
PaO2/FiO2	169.3(158.3–317.2)	191.0(134.1–232.8)	325.0(266.4–457.9) *	9.395	0.009

All values were compared with each other with * *p* < 0.05

## Discussion

NO is a selective vasodilator with clinically proven therapeutic effects and is considered the preferred treatment option for PPHN. More importantly, the U.S. FDA has only approved NO as a vasodilator for treating PPHN [21].

NO is produced by endothelial cells and causes pulmonary vasodilation via cGMP. Furthermore, iNO diffuses from alveoli to smooth muscle cells, thus leading to selective vasodilation in the pulmonary circulation and, subsequently, pulmonary vasodilation [22]. Earlier studies have elucidated that iNO can markedly improve oxygenation and reduce the use of ECMO [23, 24]. A review by Cochrane confirmed the effectiveness of iNO therapy in late preterm and term infants with neonatal respiratory failure [25]. Nonetheless, multiple studies have revealed that iNO therapy is ineffective in every infant with PPHN, with an effective rate of roughly 50% to 60%, and that infants who do not respond to iNO therapy require other vasodilators for continued treatment. The factors of non-response to iNO therapy in infants with PPHN remain poorly identified.

El-Ferzli et al. conducted a retrospective analysis of 86 cases of PPHN, including 23 cases with blood type A (18 cases treated with iNO), 21 cases with blood type B (18 cases treated with iNO), and 40 cases with blood type O (36 cases treated with iNO) [12]. Their observations elaborated that responses occurred in less than half of cases with blood type A and 90% of cases with blood types B and O after 12 h of iNO treatment and that infants of blood types B and O with PPHN more easily responded to iNO therapy than infants of blood type A, indicating that ABO blood groups may have an influence on the response to iNO therapy in infants. In our study, 90 infants with PPHN were treated with iNO alone without other vasodilators, including 24 (26.7%) infants with blood type A, 20 (22.2%) infants with blood type B, 39 (43.3%) infants with blood type O, and 7 (7.8%) infants with blood type AB. As the sample size of infants with blood type AB was small, blood type AB was excluded from further analysis. The final results showed that the highest percentage of infants who eventually responded to iNO therapy was infants with blood type O. Additionally, infants with blood type O more easily responded to iNO therapy than infants with blood type B. At the same time, there was no significant difference between infants with blood type O and infants with blood type A, as well as between infants with blood type A and infants with blood type B. Our results illustrate that ABO blood groups exerted an effect on the response to iNO therapy in infants with PPHN, where infants with blood type O more readily responded to iNO therapy than infants with blood type B, different from the findings of El-Ferzli et al. Differences in ethnicity, region, and treatment regimen may explain this difference. More cases may need to be included for further research.

Through a retrospective analysis of infants with acute hypoxemic respiratory failure (HRF) or pulmonary hypertension treated with iNO [17], McFadzean et al. observed that more time was required for PaO2/FiO2 improvement in the B/AB group compared with the O/A group. However, the proportion of cases with responses (defined as > 20% improvement in PaO2/FiO2 within 6 h) did not differ among infants of different blood groups after iNO treatment. Of note, our data unveiled differences in the efficacy of iNO among PPHN infants with different blood groups. Specifically, there was no significant difference in OI and PaO2/FiO2 values among the three groups of blood types A, B, and O before treatment. After the treatment was stopped, there were significant differences in OI and PaO2/FiO2 values, with the best oxygenation in the blood type O group and the worst in the blood type A group among the three groups. A study reported the relationship between ABO blood type and disease severity during the COVID-19 pandemic. In our study, the diagnostic criterion for severe PPHN was OI > 25 or PaO2/FiO2 ≤ 100 mmHg, and for non-severe PPHN were OI ≤ 25 or PaO2/FiO2 > 100 mmHg. The diagnostic criteria are based on OI. If the OI values were missing, PaO2/FiO2 was used as a criterion to classify the severity of PPHN [20, 21, 26–29]. The results showed that there was no significant correlation between blood type and the severity of PPHN (statistical analysis of the number of children with severe PPHN and non-severe PPHN showed *P* > 0.05). Subsequently, pairwise comparisons were carried out among the three groups, which revealed superior efficacy of iNO therapy in infants with blood type O compared to infants with blood types A and B. In contrast, there was no significant difference in efficacy between the blood type A and blood type B groups. Therefore, the efficacy of iNO therapy is relatively favorable in PPHN infants with blood type O.

Our results elucidate a correlation between ABO blood groups and responses to iNO therapy in infants with PPHN and that different blood groups affect the efficacy of NO therapy in infants with PPHN. Specifically, infants with blood type O responded most readily to iNO therapy, with the highest efficacy, among the infants with blood types A, B, and O.

This study has two main limitations. First, the sample size is small, with only 90 infants with PPHN treated with NO alone, including 83 infants with blood types A, B, and O. Therefore, more studies and data are needed for further analysis to clarify the relationship between ABO blood groups and the efficacy of iNO therapy for PPHN. Second, each collaborative unit was located in the Pearl River Delta and non-Pearl River Delta regions. The medical equipment and level vary significantly among the involved hospitals, resulting in differences in drug use and treatment effects. Although we tried to minimize the impact of bias in our analysis, some bias may also exist due to differences in the diagnosis and treatment level and the scientific research consciousness of collaborators in each involved unit. Lastly, PPHN cases with the AB blood type were not included in this study due to a small sample size. Future studies including more PPHN children with AB blood group need to be performed.

## Data Availability

All data generated or analysed during this study are included in this article. Further enquiries can be directed to the corresponding author.

## References

[1] Graves ED, Redmond CR, Arensman RM (1988). Persistent pulmonary hypertension in the neonate. Chest.

[2] Ruoss JL, Rios DR, Levy PT (2020). Updates on management for acute and chronic phenotypes of neonatal pulmonary hypertension. Clin Perinatol.

[3] Aikio O, Metsola J, Vuolteenaho R, Perhomaa M, Hallman M (2012). Transient defect in nitric oxide generation after rupture of fetal membranes and responsiveness to inhaled nitric oxide in very preterm infants with hypoxic respiratory failure. J Pediatr.

[4] Steinhorn RH (2010). Neonatal pulmonary hypertension. Pediatr Crit Care Med.

[5] American Academy of Pediatrics (2000). Committee on fetus and newborn. Use of inhaled nitric oxide. Pediatrics.

[6] Neonatal Inhaled Nitric Oxide Study Group (1997). Inhaled nitric oxide in full-term and nearly full-term infants with hypoxic respiratory failure. N Engl J Med.

[7] Roberts JD, Fineman JR, Morin FC, Shaul PW, Rimar S, Schreiber MD, Polin RA, Zwass MS, Zayek MM, Gross I, Heymann MA, Zapol WM (1997). Inhaled nitric oxide and persistent pulmonary hypertension of the newborn. The Inhaled Nitric Oxide Study Group. N Engl J Med.

[8] Wang YF, Liu CQ, Gao XR, Yang CY, Shan RB, Zhuang DY, Chen DM, Ni LM, Wang H, Xia SW, Chen C, Sun B, Collaborative Study Group for Neonatal Respiratory Diseases (2011). Effects of inhaled nitric oxide in neonatal hypoxemic respiratory failure from a multicenter controlled trial. Chin Med J (Engl).

[9] Field D, Elbourne D, Truesdale A, Grieve R, Hardy P, Fenton AC, Subhedar N, Ahluwalia J, Halliday HL, Stocks J, Tomlin K, Normand C, INNOVO Trial Collaborating Group (2005). Neonatal ventilation with inhaled nitric oxide versus ventilatory support without inhaled nitric oxide for preterm infants with severe respiratory failure: the INNOVO multicentre randomised controlled trial (ISRCTN 17821339). Pediatrics..

[10] Kinsella JP, Truog WE, Walsh WF, Goldberg RN, Bancalari E, Mayock DE, Redding GJ, deLemos RA, Sardesai S, McCurnin DC, Moreland SG, Cutter GR, Abman SH (1997). Randomized, multicenter trial of inhaled nitric oxide and high-frequency oscillatory ventilation in severe, persistent pulmonary hypertension of the newborn. J Pediatr.

[11] Weimann J, Bauer H, Bigatello L, Bloch KD, Martin E, Zapol WM (1998). ABO blood group and inhaled nitric oxide in acute respiratory distress syndrome. Lancet.

[12] El-Ferzli GT, Dreher M, Patel RP, Ambalavanan N (2012). ABO blood group is associated with response to inhaled nitric oxide in neonates with respiratory failure. PLoS One.

[13] Konduri GG, Kim UO (2009). Advances in the diagnosis and management of persistent pulmonary hypertension of the newborn. Pediatr Clin North Am.

[14] Jain A, McNamara PJ (2015). Persistent pulmonary hypertension of the newborn: advances in diagnosis and treatment. Semin Fetal Neonatal Med.

[15] Clark RH, Kueser TJ, Walker MW, Southgate WM, Huckaby JL, Perez JA, Roy BJ, Keszler M, Kinsella JP (2000). Low-dose nitric oxide therapy for persistent pulmonary hypertension of the newborn. Clinical Inhaled Nitric Oxide Research Group. N Engl J Med.

[16] Dhillon R (2012). The management of neonatal pulmonary hypertension. Arch Dis Child Fetal Neonatal Ed.

[17] McFadzean J, Tasker RC, Petros AJ (1999). Nitric oxide ABO blood group difference in children. Lancet.

[18] Thomas NJ, Shaffer ML, Willson DF, Shih MC, Curley MA (2010). Defining acute lung disease in children with the oxygenation saturation index. Pediatr Crit Care Med.

[19] Rawat M, Chandrasekharan PK, Williams A, Gugino S, Koenigsknecht C, Swartz D, Ma CX, Mathew B, Nair J, Lakshminrusimha S (2015). Oxygen saturation index and severity of hypoxic respiratory failure. Neonatology.

[20] Zhao Y, Liang L, Liu G, Zheng H, Dai L, Wang Y, Wang L, Sheng W (2021). Asphyxia and neonatal respiratory distress syndrome are independent predictors of the non-response to inhaled nitric oxide in the newborns with PPHN. Front Pediatr.

[21] Mathew B, Lakshminrusimha S (2017). Persistent pulmonary hypertension in the newborn. Children (Basel).

[22] Konduri GG (2004). New approaches for persistent pulmonary hypertension of newborn. Clin Perinatol.

[23] Van Meurs KP, Wright LL, Ehrenkranz RA, Lemons JA, Ball MB, Poole WK, Perritt R, Higgins RD, Oh W, Hudak ML, Laptook AR, Shankaran S, Finer NN, Carlo WA, Kennedy KA, Fridriksson JH, Steinhorn RH, Sokol GM, Konduri GG, Aschner JL, Stoll BJ, D'Angio CT, Stevenson DK, Preemie Inhaled Nitric Oxide Study (2005). Inhaled nitric oxide for premature infants with severe respiratory failure. N Engl J Med.

[24] Konduri GG, Solimano A, Sokol GM, Singer J, Ehrenkranz RA, Singhal N, Wright LL, Van Meurs K, Stork E, Kirpalani H, Peliowski A, Neonatal Inhaled Nitric Oxide Study Group (2004). A randomized trial of early versus standard inhaled nitric oxide therapy in term and near-term newborn infants with hypoxic respiratory failure. Pediatrics.

[25] Barrington KJ, Finer N, Pennaforte T, Altit G (2017). Nitric oxide for respiratory failure in infants born at or near term. Cochrane Database Syst Rev.

[26] Xu RD, Shi MF, Li J, Li N (2021). Severe acute respiratory syndrome coronavirus 2 virus-like particle and its application in Chinese medical research. World J Tradit Chin Med.

[27] Steinhorn RH, Fineman J, Kusic-Pajic A, Cornelisse P, Gehin M, Nowbakht P, Pierce CM, Beghetti M, FUTURE-4 study investigators (2016). Bosentan as adjunctive therapy for persistent pulmonary hypertension of the newborn: results of the randomized multicenter placebo-controlled exploratory trial. J Pediatr.

[28] Goldman AP, Tasker RC, Haworth SG, Sigston PE, Macrae DJ (1996). Four patterns of response to inhaled nitric oxide for persistent pulmonary hypertension of the newborn. Pediatrics.

[29] Ranieri VM, Rubenfeld GD, Thompson BT, Ferguson ND, Caldwell E, Fan E, Camporota L, Slutsky AS, ARDS Definition Task Force (2012). Acute respiratory distress syndrome: the Berlin Definition. JAMA.

